# Carbon dioxide and blood-feeding shift visual cue tracking during navigation in *Aedes aegypti* mosquitoes

**DOI:** 10.1098/rsbl.2022.0270

**Published:** 2022-09-28

**Authors:** Elina Barredo, Joshua I. Raji, Michael Ramon, Matthew DeGennaro, Jamie Theobald

**Affiliations:** ^1^ Department of Biological Sciences, Florida International University, Miami, FL 33199, USA; ^2^ Biomolecular Sciences Institute, Florida International University, Miami, FL 33199, USA

**Keywords:** insect vision, optomotor responses, *Aedes aegypti*, insect flight

## Abstract

Haematophagous mosquitoes need a blood meal to complete their reproductive cycle. To accomplish this, female mosquitoes seek vertebrate hosts, land on them and bite. As their eggs mature, they shift attention away from hosts and towards finding sites to lay eggs. We asked whether females were more tuned to visual cues when a host-related signal, carbon dioxide, was present, and further examined the effect of a blood meal, which shifts behaviour to ovipositing. Using a custom, tethered-flight arena that records wing stroke changes while displaying visual cues, we found the presence of carbon dioxide enhances visual attention towards discrete stimuli and improves contrast sensitivity for host-seeking *Aedes aegypti* mosquitoes. Conversely, intake of a blood meal reverses vertical bar tracking, a stimulus that non-fed females readily follow. This switch in behaviour suggests that having a blood meal modulates visual attention in mosquitoes, a phenomenon that has been described before in olfaction but not in visually driven behaviours.

## 1. Introduction

Anthropophilic mosquitoes are dangerous vectors of disease—taking a toll of over 700 000 human lives a year [1]. Mosquito control suffers from shortcomings due to insecticide resistance [2], inefficient repellents [3,4] and the lack of economical [5] and efficacious adult traps [6]. Adults emerging from aquatic stages cycle through distinct behavioural phases: nectar foraging, mating, host-seeking, resting and oviposition. To perform these tasks, they integrate visual, auditory and chemical stimuli, then make behavioural decisions according to the needs of each stage [7]. Newly emerged mosquitoes follow nectar cues for basic nutrient acquisition [8,9], which include plant semiochemicals and floral shapes and colours [9]. As they age and become reproductively mature [10,11], females increase their attraction towards human scent, relying on carbon dioxide (CO_2_) emanations, body odour, heat and likely contact cues found on the skin [7,12]. Once gravid, they are drawn towards standing water, where they lay eggs [13].

To extract relevant information from complex visual environments, animals can shift visual attention [14]. This is crucial for airborne animals, who regulate flight in response to rapidly changing scenes [15]. They may follow, avoid or ignore features depending on external factors, such as heat or odour, internal factors, such as hunger, and reproductive phase [15–17]. Visual needs vary with light habitat and activity [18], but even within a species animals may attend to varying elements of their visual landscape [16,19]. Despite the seeming importance of visual attention in host-seeking mosquitoes, there is limited knowledge about the relationship between their reproductive cycle stage and how they process information from their visual environments.

CO_2_ is a general activator of mosquito host-seeking behaviour, but there is contradicting evidence as to whether it specifically triggers visual attention [20,21] or not [22] in host-seeking *Ae. aegypti*. To address this matter, we delivered CO_2_ plumes to rigidly tethered flying mosquitoes and tested its effects on visual tracking. Next, we compared visual cue tracking between host-seeking and gravid female mosquitoes.

## 2. Methods

### (a) Insect rearing and preparation

We reared Orlando strain *Ae. aegypti* and maintained them at 25–28°C, 75% relative humidity under a 14 : 10 light–dark cycle. Eggs were hatched in hatching broth −1 l of deionized, deoxygenated water and one pellet of Tetramin fish food (catalogue no. 16152, Tetra, Melle, Germany). We sorted first-instar larvae (approx. 230 per 2 l of water) and fed them two pellets of Tetramin. Controlled larval density and food ratio guaranteed even mosquito sizes. Adult mosquitoes were fed *ad libitum* on 10% sucrose until experimentation, 6–8 days post-eclosion.

Female subjects were cold anaesthetized for a maximum of 3 min, then glued by the dorsal thorax to a tungsten rod. They rested holding a piece of tissue paper on their legs, which stops spontaneous wing beating, for 10–30 min. We suspended tethered subjects at the centre of the flight arena and removed the paper to initiate flight. Each insect was tested only once, or twice if the first trace had poor quality. We blood-fed female mosquitoes using an artificial feeder consisting of a glass tube warmed by 37°C running water. Stretched parafilm, scented by vigorous rubbing against the experimenter's arm, secured the blood (sheep blood, Fisher Scientific R54020) to the glass tube and allowed feeding through the membrane. Engorged females were separated into a cage 72 h before experiments. A custom feeder with cut transfer pipettes on the ceiling allowed capillary delivery of 10% sucrose and prevented females from laying eggs, ensuring they stayed gravid. Non-gravid females for these experiments were housed similarly.

As a reference model for our CO_2_-flight arena system, we also ran the CO_2_ experiments using the model insect, *Drosophila melanogaster* (electronic supplementary material, figure S1). Fruit fly subjects were female *D. melanogaster* from a laboratory colony, raised on a standard food medium, under a 12L : 12D cycle at 21°C and collected 4–6 days post-eclosion as done previously [19].

### (b) Tethered-flight arena and steering responses

**Figure 1.**
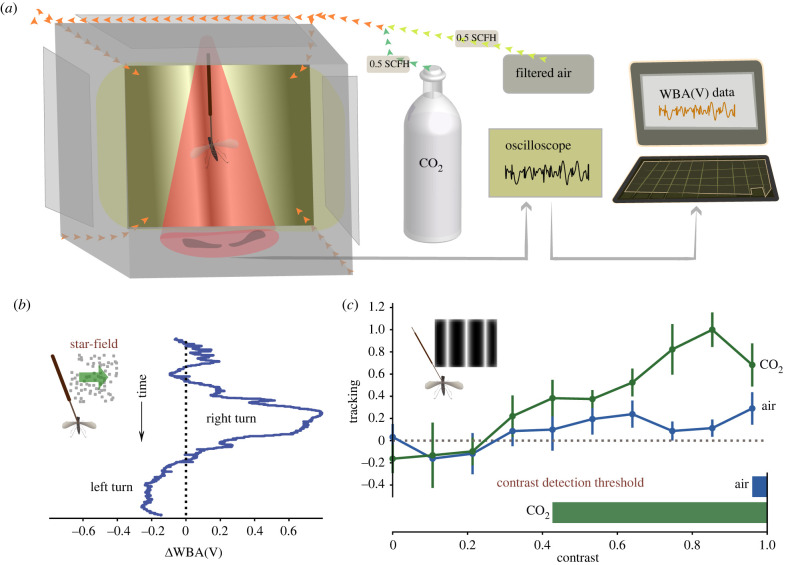
Flight arena system. (*a*) Flight arena (left) with a flying mosquito tethered under an infrared beam. CO_2_ was delivered through each corner of the front side of the cube (yellow arrows). An oscilloscope (right) recorded insect WBA as voltage (V) and frequency in hertz (Hz) used for analysis. (*b*) Representative trace of a mosquito responding to visual simulations. *Δ*WBA is calculated by subtracting the right WBA from the left. Negative and positive *Δ*WBA values suggest left and right steering attempts, respectively. (*c*) *Ae. aegypti* average tracking responses ± s.e.m. for each contrast with and without CO_2_. The bottom bar graph summarizes the minimum contrast values for which mosquito tracking responses (blue = air, *n* = 9; green = CO_2,_ *n* = 16) deviated from 0 (*p-*values < 0.05).

Our custom-built flight arena can deliver simultaneous visual and olfactory stimulation to rigidly tethered insects. Back projection screen material covers five sides of a 200 mm cube, with the back open for access, and first-surface mirrors, affixed at 45° to the sides, allow a front projector to illuminate the five faces simultaneously [23]. This covers 10.47 steradians of the visual field to a subject in the centre and displays a perspective-corrected three-dimensional scene at 360 Hz (figure 1*a*).

Within the arena, an infrared beam above the insect casts wing shadows onto two photodiodes below (figure 1*a*), which generates a voltage signal with every wing stroke. The relative amplitudes of right-wing and left-wing beats (figure 1*b*) indicate steering effort [24]. The average *Δ*WBA is calculated by subtracting the right WBA from the left for each experimental trial, divided by the total number of trials per group. Tracking values are deviations in the mean ΔWBA relative to the direction of the stimulus motion. For instance, an insect steering away from a stimulus receives a negative ΔWBA, which we call ‘anti-tracking’. For each experiment in every trial, we averaged *Δ*WBA between 1000 and 1250 ms (the shaded regions in figures below), which gave responses time to stabilize. One-tailed *t*-tests were conducted to compare the mean *Δ*WBA between groups of interest as we expected tracking to be enhanced for one of the treatments only.

### (c) Visual and olfactory stimuli

For tracking stimuli, we used star-fields of small points (113 dots sr^–1^) and frontal moving vertical bars (11 by 100°), both in open loop. For contrast sensitivity, we used frontal grating, (0.04 cycles per degree at 10 Hz) at 10 different contrasts, from 0 to 0.95 (Michelson contrast). Each mosquito saw each tracking stimulus one time for 1250 ms, in random order, in both left and right directions, interspersed with 3 s bouts of closed-loop bar fixation. Insects that failed to hold a stable bar between experiments, or failed to beat their wings throughout an experiment, were eliminated from further analysis.

Clear tubings affixed to each corner of the front screen provided CO_2_ to the arena. An airflow meter (VFA-4-SSV Dwyer Instruments Inc., IN, USA) controlled the delivery rate. We found flight was stable when CO_2_ and air were each set at 0.5 standard cubic feet per hour (SCFH), keeping the CO_2_ at 2200–2800 ppm and a working value from previous studies [25]. We monitored CO_2_ with a meter (catalogue no. CO2-100, Amprobe) placed at the back of the arena, allowing CO_2_ to be delivered continuously throughout the experiment. Control experiments were done with filtered air set at 1.0 SCFH to maintain the same airflow in all the assays.

## 3. Results

### (a) CO_2_ greatly enhances mosquito contrast sensitivity

We assessed *Ae. aegypti* responses to visual stimuli in the presence and absence of CO_2_. Without CO_2_, mosquitoes tracked only weakly at all contrasts less than 0.95. When CO_2_ was added, their contrast sensitivity increased dramatically. This difference was significant for all contrast gratings of value 0.43 or greater (air *n* = 9, CO_2_ *n* = 16, figure 1*c*). To confirm our assay was robust, we tested *Drosophila* as well and found that CO_2_ only enhanced fly contrast sensitivity at lower concentrations, in line with previous studies [26] (electronic supplementary material, figure S1A).

### (b) CO_2_ affects mosquito bar, but not star-field visual tracking

**Figure 2.**
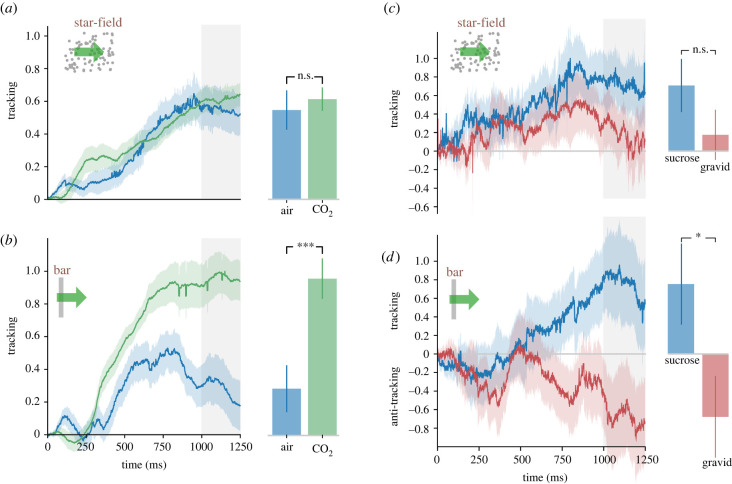
Visual attention is enhanced in the presence of CO_2_ and depends on mosquito feeding status. Normalized responses of *Ae. aegypti* female mosquitoes (*n* = 19, green = CO_2_; *n* = 17, blue = air) to a star-field (*a*) and a high-contrast bar (*b*) For the CO_2_ trials, the concentration was kept within 2200–2800 ppm. Normalized responses of gravid, blood-fed (*n* = 21, red) and non-gravid, sucrose-fed (*n* = 24, blue) *Ae. aegypti* mosquitoes tracking a star-field (*c*) and bar (*d*) in the absence of CO_2_ or air plumes. Positive *Δ*WBA values (tracking) are wing stroke deviations where mosquitoes attempt to turn towards the stimulus, while negative *Δ*WBA values represent turns in the opposite direction as the moving cue. In all line plots, the solid line represents the average response over time in milliseconds, and the shadow surrounding the line represents the s.e.m. for that treatment. Grey-shaded regions denote the time-frame compared for significance (1000–1250 ms) summarized in the bar plots ± s.e.m. Asterisks denote a significant difference in *Δ*WBA between the two treatment groups (one-tailed *t*-test).

During star-field optical flow, CO_2_ elicited little effect on mosquito tracking (air *n* = 17, CO_2_ *n* = 19, *p* = 0.32, figure 2*a*). However, mosquitoes tracked a bar much more strongly with added CO_2_ (air *n* = 17, CO_2_ *n* = 19, *p* < 0.001, figure 2*b*). When CO_2_ was delivered to *Drosophila* at the same rate as we used for mosquitoes (2200–2800 ppm), it substantially reduced fruit fly tracking during star-field optical flow as well as bar tracking, nearly disrupting *Drosophila* flight completely (air *n* = 11, CO_2_ *n* = 15, *p* < 0.001, electronic supplementary material, figure S1B,C).

### (c) The blood-fed group of mosquitoes reversed rigidly tethered bar tracking

Finally, to assess the dramatic state change that occurs in the days following a blood meal, we examined the tracking of sucrose-fed female mosquitoes, with identically aged females that had ingested a blood meal 72 h before. For star-field flow, the blood treatment resulted in a visibly reduced tracking, but that was not statistically significant at our sample size (sucrose *n* = 24, blood *n* = 21, *p* = 0.098, figure 2*c*). For moving bars, however, sucrose and blood-fed mosquitoes displayed opposite responses, with the sucrose group tracking and the blood group anti-tracking—moving opposite to the bar motion, an effect we had not seen in other experiments, (sucrose *n* = 24, blood *n* = 21, *p* = 0.015, figure 2*d*).

## 4. Discussion

*Drosophila melanogaster* has been studied under restricted flight conditions for decades [27], and we took advantage of this assay to explore the effects of CO_2_ in mosquito visual attention. Here we used contrast as a general metric of stimulus strength required for steering, star-fields as a measure for course correction and bars as a measure of target fixation, a cue that may resemble a host. Our findings align with others showing CO_2_ guides mosquitoes by enhancing object salience. When vision is impaired, as in *Ae. aegypti op1*, *op2* double mutants, mosquitoes lose tracking behaviour towards black spots with CO_2_ plumes [28]. CO_2_ is a food cue for vinegar flies as well [29] and reduced the contrast at which they followed a grating (electronic supplementary material, figure S1A). Nonetheless, the valence of fruit fly responses to CO_2_ varies depending on context (such as feeding state or CO_2_ concentration) [30], modulating the neural processing pathways that determine attractive and aversive behaviours [31].

The CO_2_ signals emitted by a vertebrate's breath reach mosquitoes at intermittent and variable doses, which triggers upwind flight to search for potential hosts [20,29,32]. Because tethered mosquitoes are flying when tested, we cannot credit CO_2_ as a general flight activator. But by enhancing bar tracking and not star-field tracking, it appears to depend on context, similar to how it enhances *Ae. aegypti* attraction for specific colours and not others [33]. We also explored if CO_2_ affected contrast sensitivity, the ability to discriminate an object from its background, a critical visual property, especially for crepuscular and nocturnal insects [34]. The eye anatomy of *Ae. aegypti* suggests that they have relatively poor resolution but may adequately detect contrast changes typical of sunset hours [35]. Since they also prefer to land on low-reflectance and high-contrast objects, and are specifically attracted to visual patterns with high vertical contrast [36], we speculate that CO_2_ alerts them to find landing targets. And although another study found no CO_2_ enhancement of tracking in tethered flight [22], that study used a free-yaw tether, in which an insect turning might rapidly affect the odour concentration the insect experiences, while the rigid tether assay here is open loop, keeping concentration constant throughout the experiment.

The multi-modal nature of host-seeking has generated extensive research into olfaction and CO_2_ sensing [12,25,37,38], but after a satisfactory blood meal, females become refractory to both host volatiles and CO_2_ [39]. Such changes in behaviour are in tune with transcriptional regulation of genes in blood-fed *Ae. aegypti* [40] and *Anopheles gambiae* [41]. In addition to olfactory genes, visual genes such as opsins are transcriptionally downregulated following blood-feeding in *An. gambiae* [42]. In behavioural assays, blood-fed *Ae. aegypti* lose interest in their hosts [13,40,43]. The bar avoidance in our blood-fed group could be a defensive strategy to avoid hosts when the priority is ovipositing, which could be further explored in free-flight assays [44]. Testing whether mosquitoes recover their bar tracking ability after laying eggs could provide additional evidence that visual attention depends on the female reproductive stage. This is the first study, to our knowledge, that presents a shift in visual attention for gravid females, and we expect the roles of CO_2_ sensing and blood-fed status in the modulation of visual attention to be largely conserved across haematophagous mosquitoes.

## Data Availability

Data are available from the Dryad Digital Repository https://doi.org/10.5061/dryad.z8w9ghxfv [45]. The data are provided in the electronic supplementary material [46].
